# Effects of probiotics on patients with Prader–Willi syndrome: a systematic review and meta-analysis of randomized controlled trials

**DOI:** 10.3389/fnut.2025.1583574

**Published:** 2025-10-22

**Authors:** Qin-Ying Toh, Yi-No Kang, Siew-Yin Chee, Hsin-Hui Chiu

**Affiliations:** ^1^Department of Pediatrics, Taipei Tzu Chi Hospital, Buddhist Tzu Chi Medical Foundation, New Taipei City, Taiwan; ^2^Cochrane Taiwan, Taipei Medical University, Taipei, Taiwan; ^3^Evidence-Based Medicine Center, Wan Fang Hospital, Taipei Medical University, Taipei, Taiwan; ^4^Institute of Health Policy and Management, College of Public Health, National Taiwan University, Taipei, Taiwan; ^5^School of Medicine, College of Medicine, Taipei Medical University, Taipei, Taiwan; ^6^Department of Pediatrics, National Taiwan University Children’s Hospital, Taipei, Taiwan

**Keywords:** syndrome of hypotonia hypomentia hypogonadism obesity, *Bifidobacterium* bifidum, *Limosilactobacillus reuteri* LR-99, psychological outcome, behavior

## Abstract

**Background:**

Prader-Willi Syndrome (PWS) involves growth, obesity, and behavioral challenges; probiotics may improve symptoms through the gut-brain axis, aiding treatment. This meta-analysis aimed to assess the impact of probiotic supplementation on individuals with PWS in terms of probiotic abundance, psycho-social outcomes, behavioral issues, and adverse events.

**Methods:**

We systematically conducted searches across PubMed, the Cochrane Central Register of Controlled Trials, EMBASE, and the Web of Science. Our study included relevant randomized controlled trials (RCTs) published before February 2025. Two independent review authors evaluated study eligibility, extracted data, and assessed the risk of bias in the included studies. Data synthesis employed a random-effects model based on heterogeneity test results and was presented as the standardized mean difference (SMD) with a 95% confidence interval (CI).

**Results:**

A total of five RCTs were included. Probiotic supplementation led to a notable increase in the abundance of the *Bifidobacterium* genus (SMD 1.21; 95% CI, 0.02 to 2.39). Notably, 12 weeks of probiotics intake demonstrated a favorable trend on social engagement (SMD −0.68; 95% CI: −1.14 to −0.21; *p* = 0.004). In contrast, probiotics did not exhibit a significant influence on behavioural problems, and the safety of probiotics consumption was assured as there was no significant increase in gastrointestinal adverse events.

**Conclusion:**

The validation of a probiotic treatment for PWS is currently an aspirational goal. Additional investigation is required to comprehensively comprehend the connection between PWS and the gut microbiome, as well as its potential ramifications for the disease phenotype.

**Systematic review registration:**

PROSPERO, CRD42023416791.

## Introduction

Prader–Willi Syndrome (PWS) is a rare genetic disorder from either paternal deletions in the chromosome 15q11–q13 region, maternal disomy, or, less frequently, imprinting defects and translocations. Clinical features in the neonatal and infantile periods include hypotonia, feeding difficulties, failure to thrive (slow growth velocity), and hypogenitalism. Later, at the age of 4–8 years, clinical manifestations, such as increased appetite, irresistible impulsive behavior toward food, and continual weight gain follow (1). In addition, intellectual impairment and many mental health problems involving social interaction and mood disorders will surface (2). These problems heighten the risk of morbid obesity (3) and unfold a long-standing fight in managing a PWS child while possessing potentially enormous economic, physical, and mental strain on caregivers in a natural setting (4). The lifelong development of obesity, sleep disorders, and endocrine issues in individuals with PWS necessitates continuous outpatient monitoring and management. It is a big challenge for clinicians to manage this multi-symptomatic disease, as there is currently no pharmacological curative agent for treatment.

In some individuals with PWS, studies show that they exhibit a decrease in growth hormone responses following stimulation tests, as well as reduced spontaneous growth hormone secretion (5) and low levels of IGF1 (6). Early treatment with growth hormone (GH) has been proposed for its benefit in linear growth, reduced body fat, motor or cognitive development, and socialization (7–9). A seminal review widely acknowledged that treating PWS with GH before the age of 2, that is, before obesity often sets in, can have significant benefits (10). However, cost constraints may actually dampen the feasibility of GH’s widespread use.

The development and progression of hyperphagia and obesity in PWS are believed to involve dysfunction in the subcortical reward circuitry and cortical inhibitory regions associated with appetite and behavioral control in the hypothalamus (11). Even with the use of growth hormone, the constant hunger sensation and food-seeking behaviors of PWS individuals could not be eradicated. Moreover, the slow metabolism and the existence of a deficiency in the production of certain hormones, including leptin and insulin, add to the difficulty of weight control. Growth hormone therapy is thus not universally effective as PWS is a complex disease involving more than just hypothalamic and pituitary dysfunction. There are also many unresolved problems, such as psychiatric, behavioral, and neurodevelopment issues, that are out of reach with GH therapy. A more accessible option using low-cost probiotics to target the gut–brain axis may shed new light on the current treatments of PWS. If this intervention aids in managing these neuropsychiatric aspects in PWS patients, it would be significantly stress-relieving for the caregivers. Simultaneously, it might be possible to decrease anti-social behaviors, promote the normalization of social interactions, and improve cognitive functions.

The gut microbiota is found to have equivalent metabolic capacity to the human liver, thus forming the link between environmental signals and its host. Its role is not limited to the gastrointestinal system; instead, it is crucial in the homeostasis of the central nervous system via the gut–microbiota–brain axis. The gut–brain bidirectional dialogue can involve various modalities such as the chemical, neuronal, or immunological pathways (12). Intestinal microorganisms produce short-chain fatty acids (SCFAs) from the fermentation of dietary indigestible fiber, which could influence neuroplasticity and microglial maturation (13, 14). Besides, bacteria could either produce neurotransmitters themselves or induce their host to produce them (15, 16), as well as generate biologically active neuropeptides (17), which acts as messengers between the gut and the brain. Communication between the microbiota and the brain through the vagus nerve is well demonstrated in animal models, where administering the Lactobacillus strain could modulate anxiety-like behaviors (18) and promote social interactions (19). Hence, microbiome dysbiosis has been reported to be associated with various neuropsychiatric conditions. Additionally, due to the established gut–brain interactions, microbiota has been suggested to be involved in obesity and metabolic health conditions (20, 21). Probiotics can display antimicrobial activity, increase the production of the intestinal mucus layer, and decrease the permeability of the intestine, enhancing its barrier function. Furthermore, they exhibit immunomodulatory effects so as to establish a “lean” host metabolism (22).

The treatments for PWS are largely behavioral, and recent research has put forward the potential for probiotics consumption as a new intervention for PWS individuals. On the basis of altering gut microbiota composition, probiotics were tested for their effect on gastrointestinal symptoms, obesity, social behaviors, and neurodevelopment in PWS. Although there is a growing number of literature supporting the beneficial use of probiotics to tackle the symptomology of PWS, our meta-analysis is the first to provide a wholesome review of the effects of probiotics on the PWS population. We postulate that this affordable and accessible intervention could stabilize the mood of patients, provide cognitive benefits, which allow for social interactions to be made, and thereby increase compliance with behavioral therapies.

## Methods

Following the Cochrane Collaboration guidelines and the Preferred Reporting Items for Systematic Reviews and Meta-Analyses (PRISMA) statement (23), we adhered to the recommended practices when conducting this systematic review. The study protocol has been officially registered with PROSPERO (CRD42023416791, an international prospective registry for systematic reviews).

### Eligibility criteria

We included randomized controlled trials (RCTs) that involved children and adults with a genetically confirmed diagnosis of Prader–Willi syndrome (PWS), which compared the effects of any form of probiotic therapy, irrespective of the form of administration, against a placebo. We planned to exclude studies based on the following criteria: (1) recruitment of both individuals with and without PWS without conducting the subgroup analysis; (2) focus on patients with PWS who also had concurrent psychiatric or gastrointestinal disorders; (3) recruitment of patients from other clinical trials or interventions; and (4) study designs limited to systematic reviews, narrative reviews, case reports, or solely *in vitro* studies.

### Search strategy, study selection process

The literature search included published articles from the following databases: PubMed, Cochrane Central Register of Controlled Trials, EMBASE, and the Web of Science, from the earliest available date up to February 2025, that compared outcomes of probiotics to placebo groups in individuals with PWS. An electronic search involving relevant keywords “Prader–Willi syndrome” and “probiotics” in free**-**text and medical subject headings were employed. Truncation was applied to capture variations of probiotic terms, and the search was conducted without restrictions on filters, publication date, age, geographic location, or language. The initial search strategy was formulated in PubMed database as outlined below: (prader willi syndrome [MeSH Terms] OR prader willi OR hhho syndrome OR H. H. H. O syndrome OR Syndrome of hypotonia hypomentia hypogonadism obesity) AND (microbiome OR Probiotics OR probiotics OR probiotic* OR Lactobacillus OR lactobacill* OR *Limosilactobacillus reuteri* OR Bifidobacterium OR bifido* OR bifidu* OR Saccharomyces OR saccharomyc* OR Streptococcus OR Enterococcus OR Escherichia OR Bacillus). Other detailed search strategy are provided in Supplementary Table S1.

We manually examined the reference lists of the included studies, as well as previously published systematic reviews and meta-analyses on the topic, to identify any additional relevant studies. Additionally, we also searched the databases of ongoing trials. Finally, we proceeded with a search update before the final publication.

The two authors independently screened each identified article’s title and abstract to eliminate duplicate entries and studies that did not meet the inclusion criteria. To prevent the exclusion of potentially pertinent articles, abstracts with unclear results were incorporated in the analysis of the full text. The full-text versions of relevant RCTs were then obtained for the additional evaluation of eligibility. The review article covered not only the usual publication aspects, such as design, study duration, randomization, blinding, interventions, measurement techniques, and follow-up, but also scrutinized the authors and institutions to ascertain whether a study was published in several articles. By verifying this information, the study mitigated the risk of overestimating results due to double-counting from a single study. Additionally, the references of the included articles were manually cross-checked.

### Data collection

Two of the authors independently extracted data from each included article using predetermined forms in Microsoft Excel 2019, which included (1) study identification, encompassing the first author’s name, year of publication, and country; (2) study design; (3) sample size and mean age of participants; (4) aim and selection criteria; (5) intervention specifics of probiotic interventions, such as frequency, and vehicle; and (6) all outcome measures. Discrepancies arising during the data extraction process were addressed through discussion between authors, with adjudication by a third reviewer (Hsin Hui-Chiu, M. D.) when necessary.

The primary outcome measures were as follows: (1) the abundance of the probiotics level, (2) psycho-social or behavioral changes, and (3) gastrointestinal adverse effects. Out of the five trials included, three focused on investigating the effects of probiotics on the abundance of desired microbiota in the stool or saliva. Two trials examined the effects of probiotics supplementation on emotional and psycho-social outcomes, while three trials explored behavioral aspects. Two trials reported gastrointestinal outcomes, specifically abdominal pain and diarrhea.

When scales and data were not provided, we looked into supplementary materials and adopted sub-scales items from the updated version of the Gilliam Autism Rating Scale, Third Edition (GARS-3) and the Child Behavior Checklist (CBCL). Psycho-social or behavioral outcomes, such as emotional responses, social engagement, and behavior problems, could be derived from the sub-scales of the two tools. In terms of emotional outcomes, the emotional response sub-scale measuring the need for reassurance during emotional outbursts, resulting from deviations in routine or triggering events, was borrowed from GARS-3. The sub-scale capturing emotional displays such as anxiety, fear, or crying was sourced from CBCL. Regarding social engagement, the subscale assessing minimal interest in others was sourced from GARS-3, while the withdrawal acts encompassing a preference for solitude or secrecy were derived from CBCL. Concerning behavioral problems’ outcome, the sub-scale addressing repetitive behaviors related to fixated interests, routines, or rituals was adopted from GARS-3, while the sub-scale encompassing thought problems involving repeated actions was sourced from CBCL. When raw data were not reported, we used^1^ to extract minimum, median, and maximum values from Figure 2 of Alyousif et al. (24), Figure 2 of Amat-Bou et al. (25), and Figure 5a of Liu and Kong (26). Then, the information obtained was used to calculate the mean change (standard deviation) in *Bifidobacterium* levels from fecal and salivary samples, transforming the findings into a standardized mean difference (SMD) for both the probiotic and placebo groups at baseline and after the intervention. At the commencement of this synthesis, the DECoMA was designated for use in standard data transformation practices (27). For instance, the SMD in gastrointestinal symptoms post-intervention was calculated using DECoMA based on the mean ± standard error reported in Table 2 by Alyousif et al. (24).

### Quality assessment

The quality of the included studies and data extraction were performed by two independent investigators using the Cochrane Collaboration Risk of Bias tool, which included domains such as the randomization generation, effect of adherence to the intervention, incomplete outcome data, appropriate measurement of outcome, and selection of the reported results (28). All discrepancies were resolved through discussion.

### Data synthesis

The effect was measured as standard mean difference (SMD) and 95% CI for the studies of probiotics compared to placebo in individuals with PWS. Data were aggregated using a random-effects model, adhering to the established recommendation for studies with diverse designs or populations (29). This approach was taken to calculate an overall effect across various contexts.

The mean change (standard deviation) of microbiota composition in fecal and saliva specimens, from baseline, was used to calculate the standardized mean difference (SMD) of the probiotics and the placebo groups at baseline and after intervention. The SMD for baseline and study timepoints at 6- and 12-weeks was determined by computing the mean change (standard deviation) for the outcomes of emotional response, psycho-social issues, and behavioral problems, as indicated by the incorporated subscales. The statistical analyses were performed using RevMan 5.4.1. Statistical heterogeneity in the results of the included studies was assessed through visual examination of forest plots, the chi-squared test with a focus on the associated *p*-value, and the calculation of the I-squared (*I*^2^) statistic. Based on feedback from external peer review, we included a subgroup analysis that was not part of the pre-registered protocol. The subgroup analysis was conducted according to two factors: age group (adults versus children) and geographic location (Western versus Eastern countries). A significant level of statistical heterogeneity was determined to exist when the *p*-value was less than 0.10 or when the *I*^2^ value exceeded 50%. During the initial phase of this synthesis, it was proposed that the funnel plot is designed to depict possible small-study effects based on data from 10 or more trials. The confidence in the main findings was further analyzed in accordance with the Grading of Recommendations Assessment, Development, and Evaluation (GRADE) framework (30).

## Results

**Figure 1:**
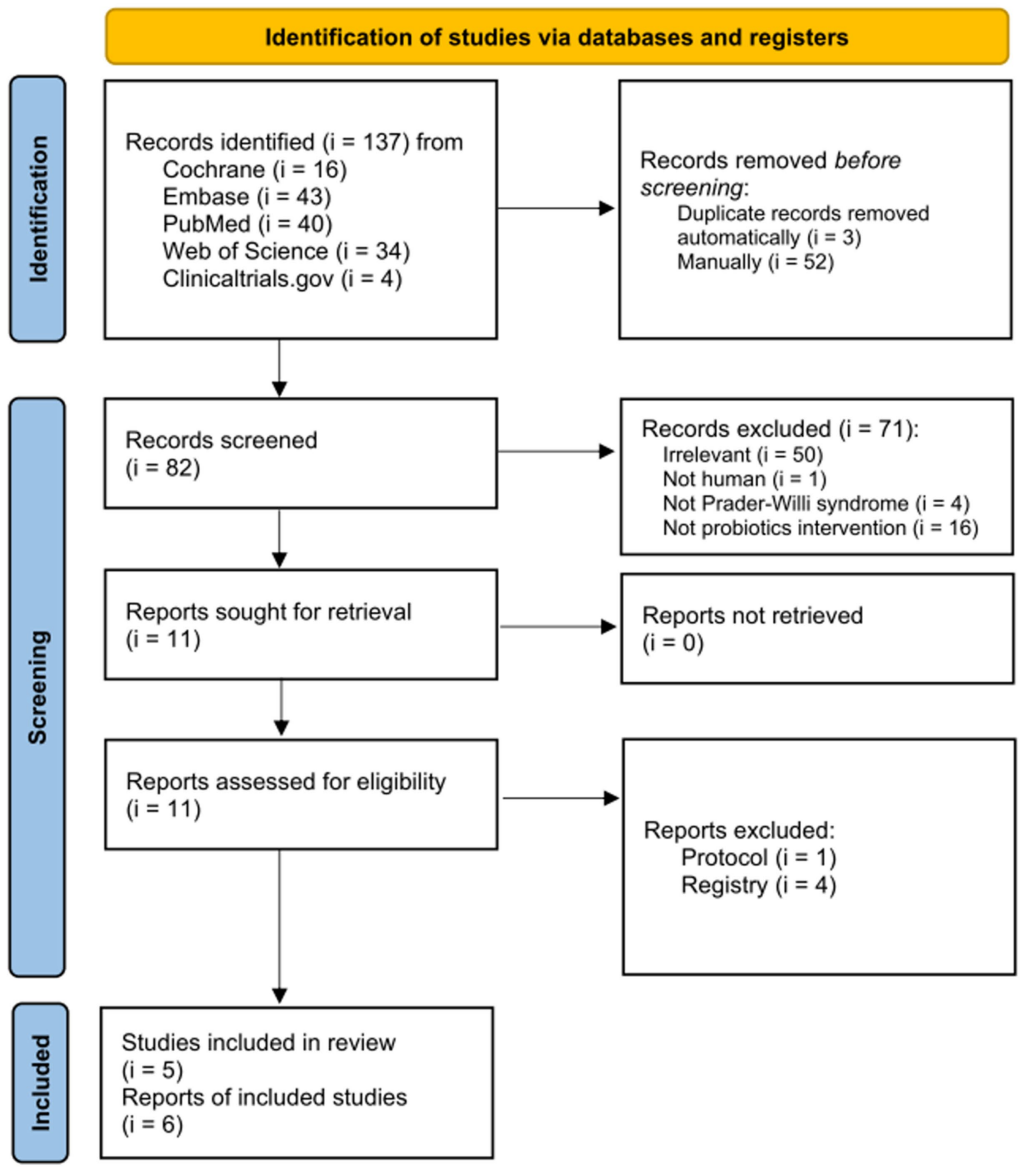
Flowchart of selection process for the evidence regarding effects of probiotics on Prader–Willi syndrome.

Database searches yielded 137 publications (Figure 1). Following the removal of duplicates, 82 out of 137 articles were initially chosen. Among these, 71 articles were excluded after screening the titles and abstracts. During the final review, studies were excluded due to non-human studies (*i* = 1), non-involvement of PWS population (*i* = 4), non-probiotic intervention (*i* = 16), and irrelevance (*i* = 50). The full texts of 11 articles were thoroughly reviewed to determine their eligibility. Consequently, the remaining five articles were included for both quantitative and qualitative analyses.

### Study characteristics

Table 1 provides a detailed overview of the key characteristics of all the studies included in the analysis. These studies, conducted in the United States, Spain, and China, were published between 2020 and 2022. Each trial followed a double-blinded placebo-controlled randomized controlled trial (RCT) design (24–26, 31, 32). In total, 239 patients were enrolled across five studies, with a portion receiving probiotic interventions ranging from 12 to 37 individuals diagnosed with PWS, and from 13 to 35 individuals in the placebo-controlled group. The focus of these studies was to investigate the effects of probiotics on individuals diagnosed with Prader–Willi syndrome (PWS). Supplementary Table S2 presents the quality evaluation of each study. Four trials (24–26, 32) used *Bifidobacterium animalis* subsp. Lactis strains and one trial (31) used *Limosilactobacillus reuteri LR-99*. The duration of probiotic supplementation for all studies lasted 12 weeks, except for Alyousif et al. (24) study, which was 4 weeks long. The product for evaluation was available in the form of capsules, gum, or tablets.

**Table 1:** Characteristics of the included randomized controlled trials.

Study	Country	Patients (Males/Females)	Mean age (range)	Body mass index	Probiotics supplement	
					Content	Frequency
Alyousif et al. (24)	United States	PS: 12 (overall) Placebo: 13 (overall)	34.9 ± 10.2 (19–56)	Overall: 30.2 ± 6.3	16 billion *B. lactis B94*	One capsule per day
Amat et al. (25)	Spain	PS: 8/9 Placebo: 6/12	10.4 ± 5 (2–19)	Overall: 1.22 ± 1.45^b^	100 mg of *Bifidobacterium animalis subsp. lactis* (BPL1, CECT8145, 10^10 colony forming units)	Once per day
ChiCTR1900022646						
Kong et al. (31)	China	PS: 37 (overall) Placebo: 34 (overall)	64.4 ± 51.0^a^ (6–264)	PS: 19.3 ± 4.58 Placebo: 19.7 ± 6.87	3 × 10^10 colony forming units (CFUs) of *Limosilactobacillus reuteri LR-99*.	One sachet twice a day
Kong et al. (32)	China	PS: 22/11 Placebo: 25/10	4.2 years old (11–16)	PS: 18.5 ± 6.4 Placebo: 19.8 ± 6.6	3 × 10^10 colony forming units (CFUs) of *Bifidobacterium animalis subsp. Lactis BL11*.	One sachet twice a day
Liu et al. (26)	China	PS: 9/8 Placebo: 10/9	59.49 ± 40.56 ^a^	PS: 23.01 ± 9.07 Placebo: 19.47 ± 5.04	3 × 10^10 colony forming units (CFUs) of *Bifidobacterium animalis subsp. Lactis BL11*.	One sachet twice a day

Kg. PS, probiotics supplementation. ^a^Month; ^b^standard deviation score.

### Effect of probiotics on *Bifidobacterium* levels

**Figure 2:**
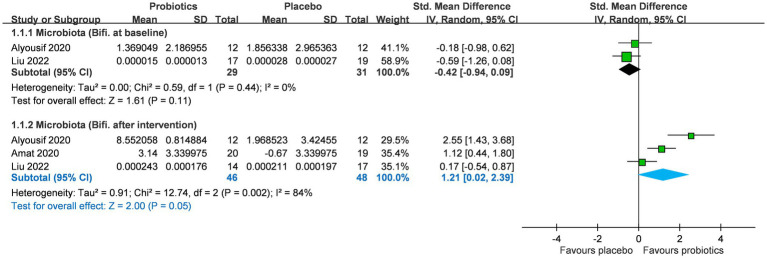
Forest plot for *Bifidobacterium* levels after intervention in microbiota. CI, confidence interval; IV, inverse variance; SE, standard error.

A total of three RCTs (*n* = 99) investigated the impact of probiotics on Bifidobacterium abundance in stool and saliva, and two studies reported Bifidobacterium abundance at baseline (Figure 2) (24–26). No significant difference was observed in *Bifidobacterium* abundance between the two groups at baseline (SMD −0.42; 95% CI: −0.94 to 0.09). After probiotics supplementation, the abundance of the *Bifidobacterium* genus was increased (SMD 1.21; 95% CI: 0.02–2.39; *p* = 0.05; I^2^ 84%; *p*-value for heterogeneity test: 0.002). This trend was not affected by age group (adults or children) and location (eastern or western countries) (Table 2).

**Table 2:** Summary of subgroup analysis by age group (adults and children) and race location.

				95% CI				Heterogeneity	
Subgroup	Trials	Sample size	Effect size	Lower	Upper	*P*-value	*I* ^2^	Change of *I*^2^	*P*-value
Microbiota									
Adults	1	24	2.55	1.43	3.68	< 0.001	NA	NA	NA
Children	2	70	0.65	−0.29	1.58	0.17	72%	12% reduction	0.06
Western countries	2	63	1.76	0.36	3.16	0.01	78%	6% reduction	0.03
Eastern country	1	31	0.17	−0.54	0.87	0.65	NA	NA	NA
Emotional problem									
Adults	–	–	–	–	–	–	–	–	–
Children	2	106	−0.04	−0.49	0.41	0.85	0%	0%	0.39
Western countries	1	35	0.29	−0.60	1.18	0.52	NA	NA	NA
Eastern country	1	71	−0.16	−0.68	0.36	0.55	NA	NA	NA
Social interaction problem									
Adults	–	–	–	–	–	–	–	–	–
Children	2	106	−0.68	−1.14	−0.21	0.004	0%	0%	0.65
Western countries	1	35	−0.50	−1.39	0.39	0.27	NA	NA	NA
Eastern country	1	71	−0.74	−1.28	−0.20	0.007	NA	NA	NA
Behavioral problem									
Adults	–	–	–	–	–	–	–	–	–
Children	3	142	0.11	−0.25	0.48	0.54	0%	0%	0.75
Western countries	1	35	0.43	−0.46	1.32	0.34	NA	NA	NA
Eastern country	2	107	0.05	−0.35	0.45	0.81	0%	0%	1

CI, confidence interval; NA, not applicable.

### Effects of probiotics on psycho-social and behavior problems

**Figure 3:**
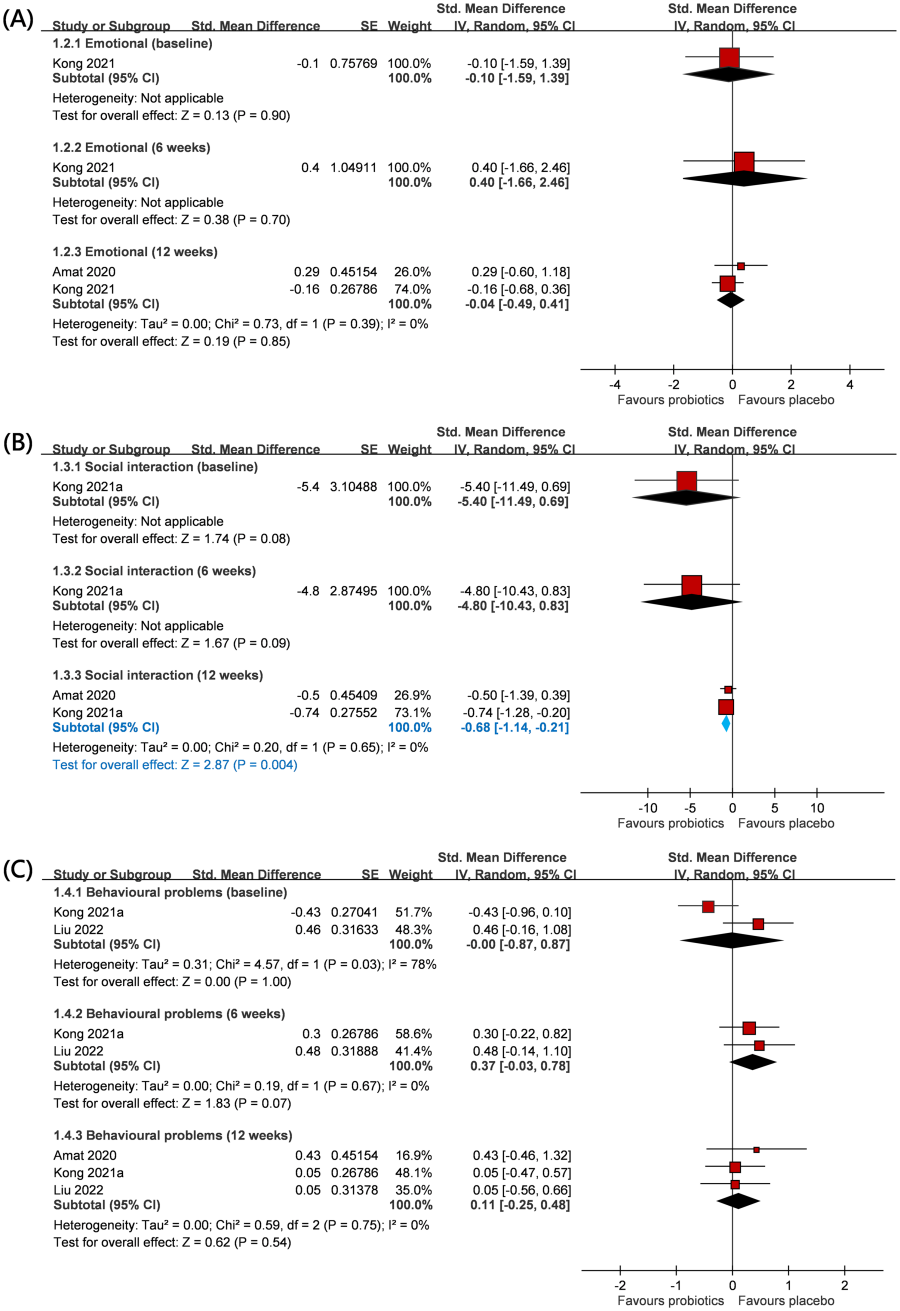
Forest plot for psycho-social and behavior outcomes: **(A)** emotional, **(B)** social interaction, and **(C)** behavioral problem. CI, confidence interval; IV, inverse variance; SE, standard error.

Psycho-social and behavioral outcomes included emotional response, social engagement, and behavioral problems based on GARS-3 and CBCL scales (Figure 3). Details have been mentioned in the Method section. With regard to emotional responses, a total of two RCTs (*n* = 43) provided relevant information on this outcome (25, 31), and one of them (25) only reported emotional responses at 12 weeks after intervention. No significant difference was observed in emotional responses between the two groups at baseline (SMD −0.10; 95% CI: −1.59 to 1.39), but the intervention effect was also not significant at study timepoints of 6-weeks (SMD 0.40; 95% CI: −1.66 to 2.46; *p* = 0.70) and 12-weeks (SMD −0.04; 95% CI: −0.49 to 0.41; *p* = 0.85; I^2^ 0%; *p*-value for heterogeneity test: 0.39). These findings did not reach statistical and clinical significance.

Regarding social engagement, a total of two RCTs (*n* = 43) provided relevant information on this outcome (25, 31), and one of them (25) only reported the social engagement outcome at 12 weeks after intervention. No significant difference was observed in social engagement between the two groups at baseline (SMD −5.40; 95% CI: −11.49 to 0.69). The intervention also did not yield a statistically significant effect at the 6-week study timepoint (SMD −4.80; 95% CI: −10.43 to 0.83; *p* = 0.09). Interestingly, after 12 weeks, probiotics showed a favorable impact on social engagement (SMD −0.68; 95% CI: −1.14 to −0.21; *p* = 0.004). Under the random effects model, I^2^ 0%; *p*-value for heterogeneity test: 0.65.

Regarding behavior problems, a total of three RCTs (*n* = 84) provided relevant information on this outcome (25, 26, 31), and one of them (25) only reported behavior problems at 12 weeks after intervention. There was no significant difference in behavior problems between the two groups at baseline (SMD −0.00; 95% CI: −0.87 to 0.87), but the intervention effect was also not significant at study timepoints of 6-weeks (SMD 0.37; 95% CI: −0.03 to 0.78; *p* = 0.07) and 12-weeks (SMD 0.11; 95% CI: −0.25 to 0.48; *p* = 0.54; I^2^ 0%; *p*-value for heterogeneity test: 0.75). These findings did not reach statistical and clinical significance. The trends were commonly observed across age groups (adults or children) and locations (eastern or western countries) (Table 2).

### Safety of probiotics

**Figure 4:**
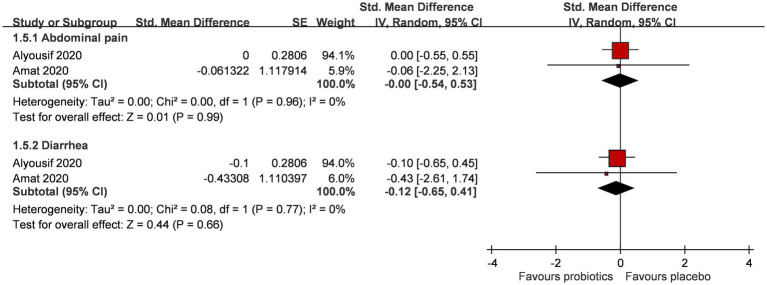
Forest plot for gastrointestinal symptoms after intervention. CI, confidence interval; IV, inverse variance; SE, standard error.

A total of two RCTs (*n* = 61) explored if probiotic supplementation had gastrointestinal adverse effects on PWS individuals (Figure 4) (24, 25). The addition of probiotic supplementation did not result in a significant increase in gastrointestinal adverse events, primarily characterized by abdominal pain (SMD 0.00; 95% CI, −0.54, 0.53; *p* = 0.99; I^2^ 0%; *p*-value for heterogeneity test: 0.96) and diarrhea (SMD -0.12; 95% CI, −0.65, 0.41; *p* = 0.66; I^2^ 0%; *p*-value for heterogeneity test: 0.77).

### Certainty of the evidence

Table 3 shows the certainty evaluation of pooled results on microbiota (specifically Bifidobacterium levels), emotional problems, social interaction issues, and behavioral problems. For microbiota, *Bifidobacterium animalis* supplementation showed a potential improvement in probiotic volume, with moderate certainty of evidence. However, for emotional, social interaction, and behavioral problems, the evidence was mostly inconclusive. Probiotics supplementation did not appear to significantly reduce emotional problems at both 6 and 12 weeks, nor did it improve social interaction or behavioral problems at 6 weeks. There was a slight improvement in social interaction problems at 12 weeks, but the overall evidence remained of low certainty due to concerns about risk of bias, study heterogeneity, and small sample sizes. Overall, while probiotics might benefit microbiota, their effects on mental health and behavioral outcomes are limited.

**Table 3:** Summary and certainty of evidence.

	Number of studies	Relative effect ^#^	Certainty of evidence	
Outcome and time point	(number of patients)	(95% CI)	(GRADE)	Comment
Microbiota (Bifi. after intervention)	3 RCTs (*n* = 94)	1.21 (0.02–2.39)	Moderate ^a,b,c,d^ ⊕ ⊕ ⊕⃝	*Bifidobacterium animalis* supplementation could improve the volume of probiotics in microbiota
Emotional problem				
6 weeks	1 RCT (*n* = 41)	0.40 (−1.66 to 2.46)	Low ^a,c,^ ⊕ ⊕ ◯⃝	Probiotics supplementation seems not reduce emotional problems in 6-week follow-up
12 weeks	2 RCTs (*n* = 111)	−0.04 (−0.49 to 0.41)	Low ^a,c,^ ⊕ ⊕ ◯⃝	Probiotics supplementation seems not reduce emotional problems in 12-week follow-up
Social interaction problem				
6 weeks	1 RCT (*n* = 41)	−4.80 (−10.43 to 0.83)	Low ^a,c,^ ⊕ ⊕ ◯⃝	Probiotics supplementation seems not reduce social interaction problems in 6-week follow-up
12 weeks	2 RCTs (*n* = 111)	−0.68 (−1.14 to −0.21)	Low ^a,c,^ ⊕ ⊕ ◯⃝	Probiotics supplementation could slightly reduce social interaction problems in 12-week follow-up
Behavioral problem				
6 weeks	2 RCTs (*n* = 82)	0.37 (−0.03 to 0.78)	Low ^a,c,^ ⊕ ⊕ ◯⃝	Probiotics supplementation seems not reduce behavioral problems in 6-week follow-up
12 weeks	3 RCTs (*n* = 107)	0.11 (−0.25 to 0.48)	Low ^a,c,^ ⊕ ⊕ ◯⃝	Probiotics supplementation seems not reduce behavioral problems in 12-week follow-up

CI, confidence interval; GRADE, Grading of Recommendations Assessment, Development and Evaluation; RCT, randomized controlled trial. ^#^Standardized mean difference. ^a^Downgrade one level due to some concerns in risk of bias. ^b^Downgrade one level due to some concerns in relatively high heterogeneity. ^c^Downgrade one level due to imprecision with small sample size. ^d^Upgrade two level due to very large effect size.

## Discussion

To the best of the authors’ knowledge, this study represents the initial meta-analysis of randomized controlled trials (RCTs) examining the impact of probiotic supplementation in individuals with PWS. Probiotics such as *Bifidobacterium* could be retained in patients with PWS after supplementation without increased risks of GI symptoms and might even decrease social interaction problems. However, there is currently no sufficient evidence to prove the beneficial effects of probiotics on behavioral and emotional problems in these patients. The effects of probiotics on body weight control also remain unclear, given the lack of anthropometry data in the included studies.

Individuals with PWS exhibit a distinct microbial profile that could potentially contribute to the manifestation of the disease phenotype. The gut microbiome also plays a crucial role in the onset and progression of various diseases, encompassing obesity, metabolic disorders, and symptoms associated with mental health. The imbalance of gut microbial may disrupt the integrity of the intestinal barrier, reduce epithelial permeability, and destabilize the overall intestinal homeostasis (33). Therefore, modulating the gut microbiota may be a promising therapeutic strategy for individuals with PWS. In the PWS group, *Bifidobacterium* exhibited reduced abundance, and previous studies have reported a decrease in *Bifidobacterium* among individuals with constipation, which resonates with PWS adults having slow transit stool form. Furthermore, individuals with constipation typically demonstrate lower levels of *Lactobacillus* and *Bacteroides* in contrast to healthy individuals (34). Hence, it is postulated that such low baseline levels of probiotic strains would result in improved retention quantity of the probiotics if taken orally.

It is notable that PWS is presented by a florid (psychotic) phenotype. During childhood, individuals with PWS may experience the emergence of mental impairment, learning difficulties, and a range of behavioral issues such as repetitive behaviors, compulsions, emotional outbursts, and skin picking (6). These behavioral challenges not only hinder academic performance but also dampen social skills and place a significant physical and mental burden on the caregivers. Probiotics supplementation emerges as a valuable option for patients with PWS if this low-cost therapy could result in a substantial enhancement of psycho-social outcomes, such as emotional, behavioral, and social interactions.

Individuals with PWS encounter specific challenges in recognizing emotions in others, accurately interpreting social interactions and empathizing with the perspectives of others. The combination of these characteristics, along with other behavioral manifestations and neurodevelopmental hindrance, collectively obstructs the capacity of PWS individuals to establish relationships and, consequently, exacerbates the feeling of loneliness (35). Our study shows that there is a promising outcome on withdrawal of social behaviors in PWS individuals after probiotics supplementation. This finding is further substantiated by a previous study showing that *L. reuteri* microbial treatment reverses social deficits via the vagus nerve and oxytocin receptors in the reward center of mouse models with Autism Spectrum Disorder (ASD) (19).

### Clinical implication

An increased abundance of *Bifidobacterium* has been linked to the alleviation of gastrointestinal symptoms such as constipation, anti-inflammatory effects, and a reduction in abdominal visceral fat—a major factor in metabolic disorders (36). Current evidence regarding the efficacy of probiotics in addressing behavioral issues in individuals with PWS remains limited. As shown in Figure 3B, probiotic-induced alterations in microbiota composition may enhance social behavior in individuals with PWS through modulation of the gut–brain axis. In the event that behavioral disorders cannot be effectively mitigated, the escalating disease severity could potentially lead to self-injury, social hazards, or criminal behavior as individuals with PWS enter adulthood.

Given the challenges of managing PWS with antipsychotics, probiotics may be a beneficial adjunct in clinical practice. In managing behavioral disturbances, collaborative care involving a psychiatrist and antipsychotic pharmacotherapy has been a viable approach. However, weight gain associated with antipsychotics is believed to stem from increased appetite, which is particularly concerning in individuals with PWS who already face challenges in controlling food-seeking behavior. Furthermore, the efficacy and outcomes of pharmacotherapy appear to vary across different PWS genotypes and may be accompanied by potential adverse effects or complex drug interactions.

There is no specific selection or approved order of medications for the treatment of behavioral problems in PWS. The need for dosage alterations also further complicates the situation. Furthermore, stimulant medications do not have the ability to evoke social interactions and communication. Apart from educational, vocational, and social functioning, the hyperphagia characteristic of PWS individuals has already profoundly affected their quality of life. In order to better support individuals with PWS, psychosocial and behavioral issues need to be clearly identified and addressed.

### Limitation

The present meta-analysis encountered several limitations. First, there was a scarcity of eligible RCTs, with most of them having a limited number of participants. The outcomes of the eligible randomized controlled trials (RCTs) also exhibited substantial heterogeneity. The assessment scales used to measure internalizing and externalizing problems lacked uniformity among the various studies included in our meta-analysis. Variability in probiotic strains, including *B. animalis* and *L. reuteri*, was observed across studies. As probiotic effects are strain-dependent, outcomes may reflect strain-specific responses influenced by inter-microbiome differences among hosts rather than overall probiotic efficacy (37). The dose–response relationship between supplementation dose and outcomes could not be determined due to the limited number of included studies. Given the small sample size with short study durations, measured in weeks, large-scale studies with extended follow-up may be necessary to evaluate the long-term stability and durability of probiotic effects.

## Conclusion

In conclusion, this systematic review provides a valuable basis for considering probiotics as an adjunct treatment for PWS patients, with some promising effects observed, particularly in the area of social participation. However, the overall positive impact appears to be relatively modest, and the evidence is still insufficient to draw definitive conclusions regarding the broader therapeutic potential of probiotics for PWS. While the studies reviewed suggest some clinical value, particularly in improving social engagement, the heterogeneity in study designs, sample sizes, and outcome measures limits the strength of these findings. Future research with larger sample sizes, more rigorous methodologies, and a broader range of outcomes is essential to better understand the full scope of benefits and to determine the clinical significance of probiotics in the management of PWS.

## Data Availability

The original contributions presented in the study are included in the article/Supplementary material, further inquiries can be directed to the corresponding author.

## Footnotes

^1^ https://plotdigitizer.com/app
