# Supplementary material for: Effects of probiotics on patients with Prader–Willi syndrome: a systematic review and meta-analysis of randomized controlled trials
Source: Front Nutr. 2025 Oct 22;12:1583574. doi: 10.3389/fnut.2025.1583574 (PMC12586056; doi:10.3389/fnut.2025.1583574)
Supplement: Supplementary file 1 [file Data_Sheet_1.PDF]

SUPPLEMENTARY FILE

Effects of probiotics on patients with Prader-Willi syndrome: a systematic review and meta-analysis of randomized controlled trials

Running title: Probiotics for Prader-Willi syndrome

Qin-Ying Toh, M.D.[1], **Yi-No Kang,** M.A.[2,3,4], **Siew-Yin Chee**, M.D. [1], **Hsin-Hui Chiu**, M.D., Ph.D., Prof.[1,5,6,*]

1. Department of Pediatrics, Taipei Tzu Chi Hospital, Buddhist Tzu Chi Medical Foundation, New Taipei City, Taiwan
2. Cochrane Taiwan, Taipei Medical University, Taipei, Taiwan
3. Evidence-Based Medicine Center, Wan Fang Hospital, Taipei Medical University, Taipei, Taiwan
4. Institute of Health Policy & Management, College of Public Health, National Taiwan University, Taipei, Taiwan
5. School of Medicine, College of Medicine, Taipei Medical University, Taipei, Taiwan
6. Department of Pediatrics, National Taiwan University Children's Hospital, Taipei, Taiwan

* Correspondence
Hsin-Hui Chiu, M.D., Ph.D.
Department of Pediatrics, Taipei Tzu Chi Hospital, Buddhist Tzu Chi Foundation
No. 289, Jianguo Rd.,Xindian,New Taipei City 231, Taiwan
ORCID: 0000-0001-8724-0661
E-mail: eliachiou@gmail.com; eliachiou@tzuchi.com.tw
Telephone number: +886-2-66289779#43231

CONTENT

Table S1 Database and search strategy

Table S2 Quality appraisal of the enrolled randomized controlled trials

Table S1 Databases and search strategy

Cochrane database (SR = 0 and Trials = 16):

prader willi OR hhho syndrome OR H.H.H.O syndrome OR Syndrome of hypotonia hypomentia hypogonadism obesity in Title Abstract Keyword AND microbiome OR Probiotics OR probiotics OR probiotic* OR Lactobacillus OR lactobacill* OR Limosilactobacillus reuteri OR Bifidobacterium OR bifido* OR bifidu* OR Saccharomyces OR saccharomyc* OR Streptococcus OR Enterococcus OR Escherichia OR Bacillus in All Text - (Word variations have been searched)

Embase (*i* = 43):

('probiotic agent'/exp OR 'probiotic' OR 'probiotic agent' OR 'probiotics' OR probiotic OR probiotics OR 'microbiome'/exp OR microbiome OR 'micro-biome' OR 'microbial biome' OR 'microbiome' OR 'microbiomes' OR 'lactobacillus'/exp OR lactobacillus OR 'betabacterium' OR 'lactobacileae' OR 'lactobacilleae' OR 'lactobacillus' OR 'lactobacteria' OR 'lactobacilli' OR lactobacill* OR 'limosilactobacillus reuteri' OR 'bifidobacterium'/exp OR bifidobacterium OR 'bifidobacterium' OR bifido* OR 'lactobacillus bifidus'/exp OR 'lactobacillus bifidus' OR 'bifidus, lactobacillus' OR 'saccharomyces'/exp OR saccharomyces OR 'saccharomyces' OR saccharomyc* OR 'streptococcus'/exp OR streptococcus OR 'streptococceae' OR 'streptococcus' OR 'streptococcus species' OR 'streptococcal component' OR 'streptococci' OR 'enterococcus'/exp OR enterococcus OR 'enterococcus' OR 'escherichia'/exp OR escherichia OR 'escherichia' OR 'bacillus'/exp OR bacillus OR 'bacillus') AND ('prader willi syndrome'/exp OR 'prader willi' OR 'prader willi syndrome' OR 'prader-willi syndrome' OR 'hhho syndrome' OR 'labhart willi prader syndrome' OR 'prader labhart willi syndrome' OR 'prader willi labhart syndrome' OR 'willi prader labhart syndrome' OR 'willi prader lalhart syndrome' OR 'syndrome of hypotonia hypomentia hypogonadism obesity' OR 'h.h.h.o. syndrome')

PubMed (*i* = 40):

(prader willi syndrome[MeSH Terms] OR prader willi OR hhho syndrome OR H.H.H.O syndrome OR Syndrome of hypotonia hypomentia hypogonadism obesity) AND (microbiome OR Probiotics OR probiotics OR probiotic* OR Lactobacillus OR lactobacill* OR Limosilactobacillus reuteri OR Bifidobacterium OR bifido* OR bifidu* OR Saccharomyces OR saccharomyc* OR Streptococcus OR Enterococcus OR Escherichia OR Bacillus)

Web of Science (*i* = 34)

ALL=(prader willi OR hcho syndrome OR H.H.H.O syndrome OR Syndrome of hypotonia hypodontia hypogonadism obesity) AND ALL=(microbiome OR Probiotics OR probiotics OR probiotic* OR Lactobacillus OR lactobacill* OR Limosilactobacillus reuteri OR Bifidobacterium OR bifido* OR bifidu* OR Saccharomyces OR saccharomyc* OR Streptococcus OR Enterococcus OR Escherichia OR Bacillus)

i, numbers of reference found; SR, systematic review.

Table S2 Quality appraisal of the enrolled randomized controlled trials

Trial	D1	D2	D3	D4	D5	Overall
**Microbiota (Bifi. after intervention)**						
Alyousif 2020	Low risk	Low risk	Some concerns	Low risk	Low risk	Some concerns
Amat 2020	Low risk	Some concerns	Low risk	Low risk	Low risk	Some concerns
Liu 2022	Low risk	Some concerns	Low risk	Low risk	Low risk	Some concerns
**Emotional (6 weeks)**						
Kong 2021	Low risk	Low risk	Low risk	Low risk	Low risk	Low risk
Emotional (12 weeks)						
Amat 2020	Low risk	Some concerns	Low risk	Low risk	Low risk	Some concerns
Kong 2021	Low risk	Low risk	Low risk	Low risk	Low risk	Low risk
**Social interaction (6weeks)**						
Kong 2021	Low risk	Low risk	Low risk	Low risk	Low risk	Low risk
Amat 2020	Low risk	Some concerns	Low risk	Low risk	Low risk	Some concerns
**Social interaction (12weeks)**						
Kong 2021	Low risk	Low risk	Low risk	Low risk	Low risk	Low risk
**Behavioural (6weeks)**						
Kong 2021	Low risk	Low risk	Low risk	Low risk	Low risk	Low risk
Liu 2021	Low risk	Some concerns	Some concerns	Low risk	Low risk	Some concerns
**Behavioural (12weeks)**						
Amat 2020	Low risk	Some concerns	Low risk	Low risk	Low risk	Some concerns
Kong 2021	Low risk	Low risk	Low risk	Low risk	Low risk	Low risk
Liu 2022	Low risk	Some concerns	Some concerns	Low risk	Low risk	Some concerns
**Abdominal pain**						
Alyousif 2020	Low risk	Low risk	Low risk	Low risk	Low risk	Low risk
Amat 2020	Low risk	Some concerns	Low risk	Low risk	Low risk	Some concerns
**Diarrhea**						
Alyousif 2020	Low risk	Low risk	Low risk	Low risk	Low risk	Low risk
Amat 2020	Low risk	Some concerns	Low risk	Low risk	Low risk	Some concerns

D1: randomization process; D2: effect adhering to intervention; D3: missing outcome data; D4: measurement of the outcome; D5: selection of the reported result.
