# Supplementary figures and images for: Effects of probiotics on patients with Prader–Willi syndrome: a systematic review and meta-analysis of randomized controlled trials
Source: Front Nutr. 2025 Oct 22;12:1583574. doi: 10.3389/fnut.2025.1583574 (PMC12586056; doi:10.3389/fnut.2025.1583574)

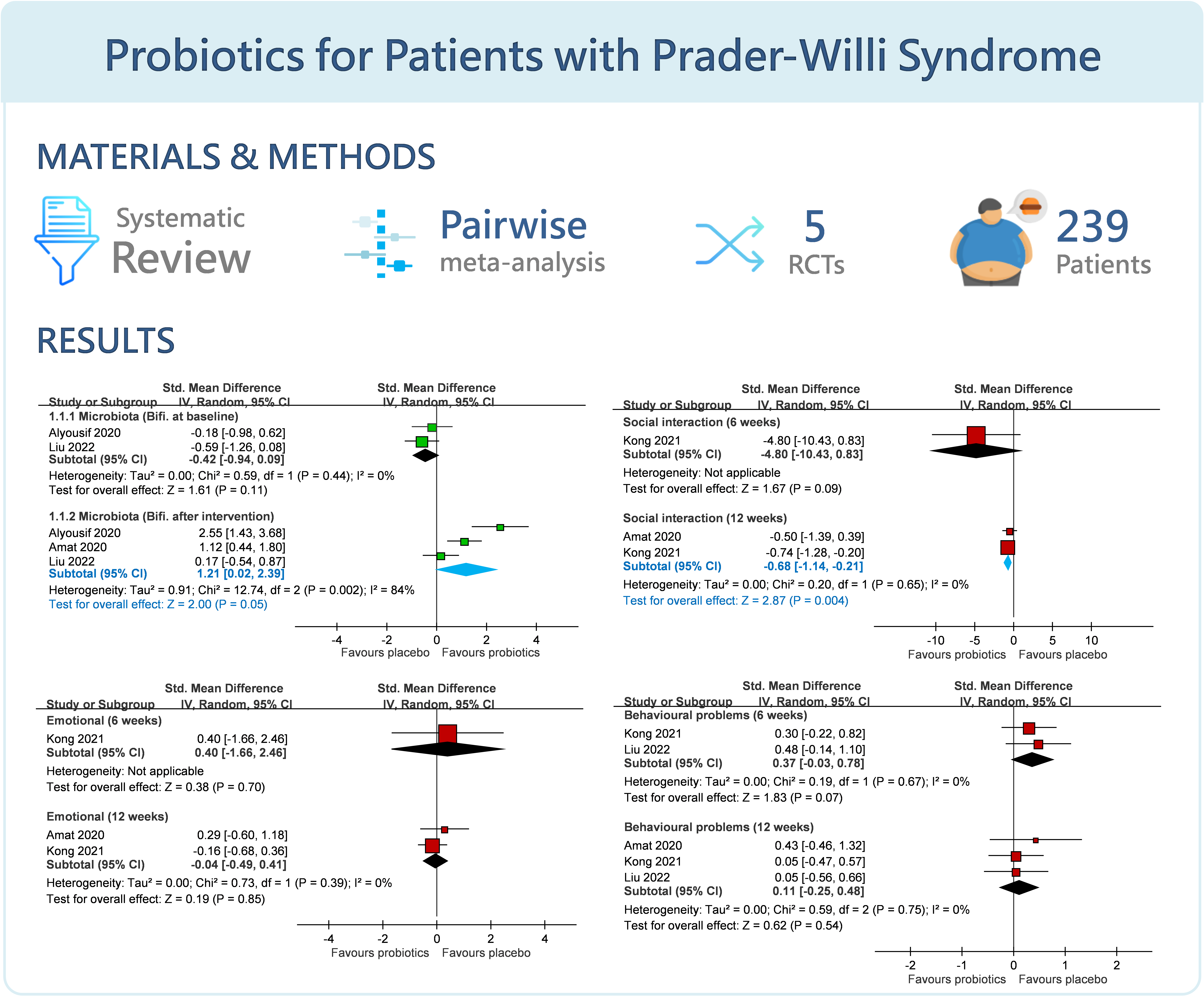

Supplement: Supplementary file 2 [file Image_1.TIF]
